# Mass Azithromycin Distribution to Prevent Childhood Mortality: A Pooled Analysis of Cluster-Randomized Trials

**DOI:** 10.4269/ajtmh.18-0846

**Published:** 2019-01-02

**Authors:** Catherine E. Oldenburg, Ahmed M. Arzika, Abdou Amza, Teshome Gebre, Khumbo Kalua, Zakayo Mrango, Sun Y. Cotter, Sheila K. West, Robin L. Bailey, Paul M. Emerson, Kieran S. O’Brien, Travis C. Porco, Jeremy D. Keenan, Thomas M. Lietman

**Affiliations:** 1 Francis I Proctor Foundation, University of California, San Francisco, San Francisco, California;; 2 Department of Ophthalmology, University of California, San Francisco, San Francisco, California;; 3 The Carter Center, Niamey, Niger;; 4 Programme FSS/Université Abdou Moumouni de Niamey, Programme National de Santé Oculaire, Niamey, Niger;; 5 The Carter Center, Addis Ababa, Ethiopia;; 6 Blantyre Institute for Community Outreach and the College of Medicine, University of Malawi, Blantyre, Malawi;; 7 National Institute for Medical Research, Dar es Salaam, Tanzania;; 8 The Dana Center, Johns Hopkins University School of Medicine, Baltimore, Maryland;; 9 The London School of Tropical Hygiene and Medicine, London, United Kingdom;; 10 The International Trachoma Initiative, Decatur and Emory University, Atlanta, Georgia

## Abstract

Mass drug administration (MDA) with azithromycin may reduce under-5 child mortality (U5M) in sub-Saharan Africa. Here, we conducted a pooled analysis of all published cluster-randomized trials evaluating the effect of azithromycin MDA on child mortality. We pooled data from cluster-randomized trials randomizing communities to azithromycin MDA versus control. We calculated mortality rates in the azithromycin and control arms in each study, and by country for multisite studies including multiple countries. We conducted a two-stage individual community data meta-analysis to estimate the effect of azithromycin for prevention of child mortality. Three randomized controlled trials in four countries (Ethiopia, Malawi, Niger, and Tanzania) were identified. The overall pooled mortality rate was 15.9 per 1,000 person-years (95% confidence interval [CI]: 15.5–16.3). The pooled mortality rate was lower in azithromycin-treated communities than in placebo-treated communities (14.7 deaths per 1,000 person-years, 95% CI: 14.2–15.3 versus 17.2 deaths per 1,000 person-years, 95% CI: 16.5–17.8). There was a 14.4% reduction in all-cause child mortality in communities receiving azithromycin MDA (95% CI: 6.3–21.7% reduction, *P* = 0.0007). All-cause U5M was lower in communities receiving azithromycin MDA than in control communities, suggesting that azithromycin MDA could be a new tool to reduce child mortality in sub-Saharan Africa. However, heterogeneity in effect estimates suggests that the magnitude of the effect may vary in time and space and is currently not predictable.

## INTRODUCTION

More than 700 million doses of azithromycin have been distributed to trachoma-endemic districts as part of trachoma control.^1^ Annual mass drug administration (MDA) with azithromycin dramatically reduces the prevalence of *Chlamydia trachomatis*, the causative organism of trachoma.^2,3^ Collateral effects of azithromycin MDA may include reduction in infectious burden, including malaria parasitemia,^4,5^ upper respiratory infection,^6^ and diarrhea,^7^ and improving nutritional status.^8–10^ Unintended but beneficial effects of mass azithromycin distribution may lead to improvements in child health at the population level that extend beyond those for trachoma control.

In a cluster-randomized trial of azithromycin MDA for trachoma control, communities receiving azithromycin had significantly decreased all-cause child mortality compared with communities receiving no treatment.^11^ In a subsequent three-country study designed specifically to assess the efficacy of azithromycin MDA for reduction in child mortality, biannual azithromycin MDA to preschool children decreased all-cause child mortality by 13.5% compared with communities receiving placebo.^12^ However, the efficacy of mass azithromycin for the reduction in mortality differed substantially across the sites included in the trial, although there was no statistically significant difference between the countries. The strongest effect by far was seen in the Niger site, which had the highest baseline child mortality rate, the greatest number of child-years of observations, the lowest loss to follow-up, and the highest coverage with MDA. As such, the greatest number of events to report on arose from Niger.

Here, we assess the sum of the evidence of the effect of azithromycin MDA on child mortality in a pooled analysis of cluster-randomized trials. We further sought to address sources of heterogeneity of effects of the relationship between azithromycin MDA and child mortality.

## METHODS

To identify trials, we searched Medline using the key words “mortality” and “azithromycin or zithromax” from inception through November 22, 2018. Titles and abstracts were reviewed by the C. E. O., and studies that met the inclusion criteria were included in the pooled analysis. Trials were eligible for inclusion in the pooled analysis if they randomized communities to azithromycin MDA strategies and reported all-cause child mortality as a prespecified outcome. Individually randomized studies were excluded as the research question was focused on community-level mass azithromycin distribution for prevention of child mortality. Studies reporting any azithromycin indication (e.g., for trachoma control or not) were eligible for inclusion. Studies were eligible for inclusion if they prespecified the mortality outcome and the statistical analysis plan a priori. The outcome of interest was all-cause mortality in children aged less than 5 years. We used individual data from each trial to estimate the effect of azithromycin on all-cause mortality in children up to 59 months of age, even if the original study reported a wider age range. Only children in an age range that was eligible for treatment were included in the analysis.

### Characteristics of included studies.

Characteristics of studies included in the pooled analysis are summarized in Table 1.

**Table 1:** Studies included in the pooled analysis

Country	Study	Years of study	Country child mortality rate,* per 1,000 live births	Child mortality rate* in control arm per 1,000 person-years	Number of communities	Azithromycin intervention	Control intervention
Ethiopia	TANA	2006–2007	103.5	8.3	48	Annual, biannual or quarterly mass azithromycin	Delayed mass azithromycin distribution
Niger	PRET	2010–2013	116.1	35.3	48	Biannual mass azithromycin to children < 12 years	Annual mass azithromycin to the entire community
Niger	MORDOR	2015–2017	93.2	27.5	594	Biannual mass azithromycin to children aged 1–59 months	Biannual placebo to children aged 1–59 months
Malawi	MORDOR	2015–2017	57.1	9.6	304	Biannual mass azithromycin to children aged 1–59 months	Biannual placebo to children aged 1–59 months
Tanzania	MORDOR	2015–2017	57.8	5.5	614	Biannual mass azithromycin to children aged 1–59 months	Biannual placebo to children aged 1–59 months

MORDOR = *Macrolide Oraux pour Réduire les Décès avec un Oeil sur la Résistance*; PRET = Partnership for the Rapid Elimination of Trachoma; TANA = Trachoma Amelioration in Northern Amhara.

* Child mortality rates as estimated by United Nations International Children’s Emergency Fund (UNICEF) are expressed in number of deaths per 1,000 live births; mortality rates as estimated by the control arm in each trial are mortality rates per 1,000 person-years. Mortality rates per 1,000 person-years estimate the number of deaths per 1,000 persons per year, whereas mortality rates per 1,000 live births are the probability (per 1,000 live births) of a child dying before his or her fifth birthday (e.g., over a 5-year period).

The Trachoma Amelioration in Northern Amhara (TANA) study was a four-arm community-randomized trial of azithromycin MDA strategies for trachoma control, including annual (ages ≥ 1 year), biannual (ages ≥ 1 year), quarterly (ages 1–9 years), and delayed treatment.^11,13^ Trachoma Amelioration in Northern Amhara took place in Amhara, Ethiopia, and mortality results were reported for the period from 2006 to 2007. Mortality was measured via a door-to-door census annually before the annual treatment phase. Communities were followed for 12 months, and all-cause child mortality was a prespecified secondary outcome. In this analysis, we restricted mortality data only to children aged 12–59 months for consistency with other studies. Children less than 12 months of age were not treated in TANA and, thus, were not included in this analysis.

The Partnership for the Rapid Elimination of Trachoma (PRET) study was a three-country study of annual azithromycin MDA (ages ≥ 6 months) compared with only biannual treatment of children (ages 6 months–12 years). Mortality data were available and prespecified as a secondary outcome for the Niger study.^14,15^ In Niger, communities were randomized in a 1:1:1:1 ratio to annual azithromycin MDA with an 80% coverage target, annual azithromycin MDA with a 90% coverage target, biannual azithromycin MDA with 80% coverage, or biannual azithromycin MDA with 90% coverage. Mortality was determined by a door-to-door census conducted before each annual treatment phase. The communities were followed for 36 months. Children aged 6–59 months were included in the mortality analysis.

The *Macrolide Oraux pour Réduire les Décès avec un Oeil sur la Résistance* (MORDOR) study was a placebo-controlled community-randomized trial conducted in Malawi, Niger, and Tanzania.^12^ The communities were randomized in a 1:1 ratio to biannual azithromycin MDA or biannual placebo distribution to preschool children (ages 1–59 months). Mortality was measured via a door-to-door census conducted before each treatment phase. Communities were followed for a 24-month period, and the primary prespecified outcome was all-cause mortality as determined by the census. Children aged 1–59 months were included in the mortality analysis.

Each included study was reviewed and approved by the appropriate ethical committee, including the Committee on Human Research at the University of California, San Francisco (TANA, PRET, MORDOR); the Ethiopian Science and Technology Commission (TANA); and the Institutional Review Boards at Emory University (TANA, MORDOR), the College of Medicine, University of Malawi, Blantyre (MORDOR), the Niger Ministry of Health (PRET, MORDOR), the Tanzanian National Institute for Medical Research (MORDOR), the London School of Hygiene and Tropical Medicine (MORDOR), and Johns Hopkins University School of Medicine (MORDOR). The pooled analysis used de-identified community-level aggregate data and did not require additional ethical review.

### Statistical methods.

We conducted a two-stage individual community data meta-analysis using community-level data from each study. All analyses were conducted at the community level (the level of randomization) to account for the cluster-randomized nature of the studies and were intention-to-treat. For the first stage, we calculated incidence rate ratios (IRRs) for each country from each study separately using a negative binomial model. We then used the individual study IRRs and their variances and pooled the estimates into a single summary measure using a random effects model, with a separate random effect for each country from each study (five random effects total) using the *metafor* package in R (The R Foundation for Statistical Computing, Vienna, Austria). A restricted maximum likelihood (REML) estimator was used to estimate τ2. We estimated heterogeneity across trials with the *I*2 statistic. The primary analysis considered each country and each trial separately to assess heterogeneity by country (e.g., including both geography and background mortality rates at the time the study was conducted). To estimate the pooled risk difference, we calculated study-specific incidence rates in the azithromycin and control arms and used random effects meta-analysis with an REML estimator to calculate the pooled estimate. We assessed heterogeneity of effect estimates by country of origin and the background under-5 child mortality rate (U5MR) as determined by the mortality rate in the control arm of the study. We built separate random effects models for each moderator of interest using the *metafor* command in R. As a sensitivity analysis, we repeated the primary analysis excluding one study in which all communities received at least annual azithromycin MDA (e.g., excluding the PRET-Niger study).^16^ All analyses were conducted in R.

## RESULTS

A total of 449 unique titles and abstracts were identified and reviewed. Reasons for exclusion included that the study did not meet the inclusion criteria (e.g., was nonrandomized or individuals randomized, in adults; did not evaluate azithromycin; or did not report mortality, *N* = 194); was an editorial, review, case report, or other non-empirical report (*N* = 190); or was a nonhuman study (*N* = 62). We identified three cluster-randomized trials undertaken in 1,608 communities from four countries (e.g., Ethiopia, Malawi, Niger, and Tanzania) that met the inclusion criteria.^11,12,16^ Azithromycin interventions included annual, biannual, and quarterly MDA to the whole community or to children only (Table 1). Control communities included distribution of matching placebo (MORDOR), delayed azithromycin MDA (TANA), and annual azithromycin MDA (standard of care for trachoma-endemic communities, PRET). Across all studies and all sites, a total of 5,486 deaths were observed over 344,905 person-years (mortality rate 15.9 per 1,000 person-years, 95% CI: 15.3–16.1). In azithromycin-treated communities, the mortality rate was 14.4 per 1,000 person-years (95% CI: 13.9–15.0), compared with 17.0 per 1,000 person-years (95% CI: 16.4–17.6) in untreated communities.

**Figure 1.**
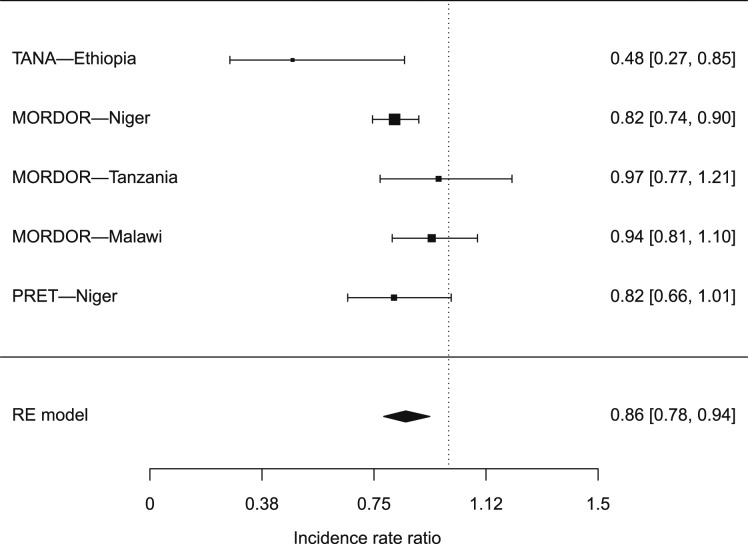
Forest plot of incidence rate ratios for studies included in the pooled analysis. Studies evaluated child mortality rates in communities receiving mass drug administration with azithromycin compared with control (placebo distribution in *Macrolide Oraux pour Réduire les Décès avec un Oeil sur la Résistance* [MORDOR], annual azithromycin in Partnership for the Rapid Elimination of Trachoma [PRET], and delayed treatment in Trachoma Amelioration in Northern Amhara [TANA]). Data were included for children aged 1–59 months in MORDOR, 6–59 months in PRET, and 12–59 months in TANA.

Across all studies and countries, in a random effects model taking into account between-study heterogeneity, there was a 14.4% reduction in mortality in communities that received azithromycin MDA versus control communities (pooled IRR: 0.856, 95% CI: 0.783–0.937, *P* = 0.0007; Figure 1). There was moderate heterogeneity across studies (*I*^2^ = 22.6%, *P* = 0.11). The pooled incidence rate difference was 2.9 fewer deaths per 1,000 person-years in azithromycin-treated communities than in placebo communities (95% CI: −5.6 to −0.3 deaths per 1,000 person-years, *P* = 0.03). The results were robust to exclusion of the PRET-Niger study, in which the control arm included annual mass azithromycin distribution (pooled IRR: 0.865, 95% CI: 0.770–0.972, *P* = 0.02).

**Figure 2.**
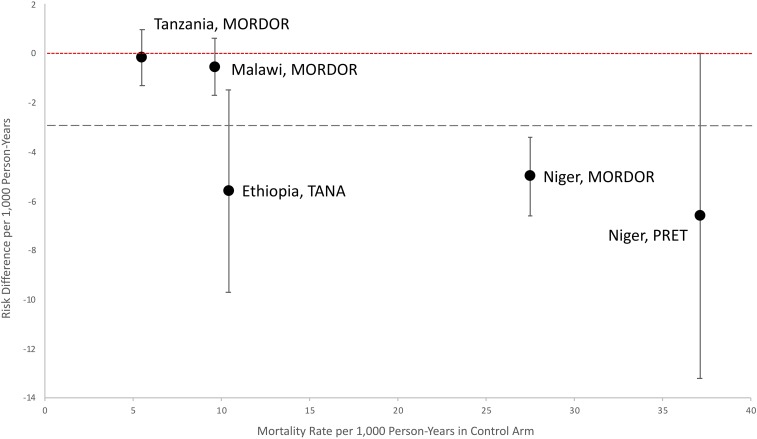
Risk differences and 95% confidence intervals for rate of mortality in azithromycin-treated communities compared with placebo-treated communities. The red dotted line with short dashes indicates 0 deaths averted per 1,000 person-years (no effect). The gray dotted line with long dashes indicates the pooled effect estimate (−2.9 deaths averted per 1,000 person-years). MORDOR = *Macrolide Oraux pour Réduire les Décès avec un Oeil sur la Résistance*; PRET = Partnership for the Rapid Elimination of Trachoma; TANA = Trachoma Amelioration in Northern Amhara. This figure appears in color at www.ajtmh.org.

We explored several sources of heterogeneity, including country and background child mortality rate. Study sites with a higher mortality rate in the placebo arm tended to have greater absolute differences in the rate of child mortality between the azithromycin and the placebo arm (Figure 2). However, in a meta-regression model, there was no significant effect modification by mortality rate in the placebo arm (*P* = 0.12), although in general the effect sizes increased with increasing baseline mortality rate. Effect modification by country of origin was not statistically significant (*P* = 0.06).

## DISCUSSION

Across three studies in four countries that met our inclusion criteria, under-5 child mortality (U5M) was 14% lower in communities receiving azithromycin MDA than in control communities, although effect estimates between studies showed some degree of heterogeneity. It is tempting to hypothesize that heterogeneity was driven primarily by the background mortality rate, but there was no significant difference by child mortality rate or country of the study site. However, detection of effect modification is difficult in studies of rare outcomes such as child mortality.^17^ Even in this large pooled analysis, detection of statistically significant effect modification may not be possible. The potential absolute number of lives saved with azithromycin MDA will be greater in scenarios with more child mortality events (higher baseline U5MR, or areas with an outbreak or epidemic of disease amenable to treatment with azithromycin), even if the proportion is similar between settings overall. In general, studies conducted in communities with higher baseline mortality had larger effect sizes, indicating that azithromycin MDA may be the most efficacious in high child mortality burden settings. Child mortality continues to decline over time in many regions of sub-Saharan Africa.^18^ It is possible that geographic indication of azithromycin for child mortality reduction could differ across space and time.

Although the reason for the differences in efficacy of azithromycin MDA in each study setting is unclear, there are several potential reasons why efficacy may differ. Under-5 child mortality is not normally distributed and the underlying causality may vary greatly in time and space. If the underlying mortality driver is periodic outbreaks or epidemics of viral meningitis or rotavirus, for example, the effect of azithromycin MDA is likely to be far less than in a setting in which bacterial respiratory tract infection, bacterial diarrhea, and/or malaria is the driver of high mortality. In general, the largest effect sizes were seen in countries with larger mortality rates in the placebo arm and higher country-level mortality rates, larger samples, and higher coverage with azithromycin MDA. Child mortality is a rare event even in high-burden settings. In low- and medium-burden settings where child mortality is a very rare event, absolute effect sizes will necessarily be small, and there will be limited power to detect relative differences. Differences in causes of death by study site or time point may affect the efficacy of azithromycin, for example, if causes of death in one location are more often an infectious agent that is susceptible to azithromycin than others. Differences in background antibiotic use and susceptibility to macrolides of bacterial pathogens may also contribute to heterogeneity. Azithromycin distribution may have reduced efficacy for prevention of mortality in areas that have increased macrolide resistance. It is possible that underlying pathogens that contributed to mortality and morbidity or their antibiotic susceptibilities differed across geography and time in the included studies.

Some heterogeneity in study-specific effects is likely because of the design of individual studies included in this analysis. The cluster-randomized trials that were designed for trachoma endpoints included in this analysis did not include a placebo control group.^11,16^ This could cause bias in outcome assessment, given that census activities were tied to treatment activities. The biggest anticipated threat would be that fewer deaths would be discovered in untreated communities because census/treatment teams would not have contact with these communities during the study. Such bias does not seem to have been present, however, because a protective effect of azithromycin MDA on mortality was observed even in studies with an untreated control group. However, if deaths were missed in the untreated arm of these studies, these results could be an underestimate of the true effect of azithromycin MDA.

Trachoma studies have demonstrated that azithromycin MDA leads to selection for macrolide resistance in some organisms, including *Streptococcus pneumoniae*^19–22^ and *Escherichia coli*.^23–25^ However, resistance selection in the most commonly used therapeutic antibiotic classes for childhood infection in sub-Saharan Africa (penicillins and sulfamethoxazole) has generally not been found.^6,19,21^ Trachoma programs distribute millions of doses of azithromycin annually,^26^ of which approximately 15% are administered to children of ages 6 months to 5 years. Health districts are targeted for a predetermined number of years (1, 3, or 5) determined by the baseline prevalence of clinical signs of trachoma children.

Programmatic use of azithromycin MDA for the prevention of child mortality could introduce additional macrolide selection pressure if it were not similarly time limited. Therefore, understanding the drivers of heterogeneity in treatment effects in time and space is essential for targeting azithromycin interventions to regions that would benefit the most to minimize resistance. The results of the present analysis suggest that consideration of baseline child mortality rates (which differ over time and with geography), the size of the population targeted, and the anticipated coverage rate may be important when deciding where to implement azithromycin MDA to reduce U5M.

The results of this study must be interpreted in the context of several limitations. As previously described, not all studies included a placebo arm. Treatment and control strategies differed across studies, which likely introduced additional heterogeneity. Detection of heterogeneity in treatment effects (effect modification) with rare outcomes is difficult even in large trials.^17^ Although individual study results differed qualitatively across space and time, there were no statistically significant effect modifiers. To date, only a limited number of studies have evaluated the efficacy of azithromycin for child mortality. All studies included in this pooled analysis were conducted by the same group of investigators. If azithromycin MDA is scaled up in the context of child mortality reduction programs, continual evaluation of the effectiveness of the intervention will be essential to determine when to stop.

The results of this analysis demonstrate a significant protective effect of azithromycin MDA to preschool children on all-cause mortality in studies reported over a nearly 10-year period. There was some heterogeneity in effect estimates, likely reflecting both changes over time and space, and differences in study design. Although the precise mechanism of action of azithromycin for prevention of child mortality remains unknown, these results indicate a potentially important role of azithromycin MDA for mortality reduction in sub-Saharan Africa.
